# The relationship between insomnia and complex diseases—insights from genetic data

**DOI:** 10.1186/s13073-019-0668-0

**Published:** 2019-08-29

**Authors:** Enda M. Byrne

**Affiliations:** Institute for Molecular Bioscience, The University of Queensland, Carmody Road, Brisbane, QLD 4072 Australia

## Abstract

Insomnia is a common condition whose pathophysiology is poorly understood. Large genetic studies have provided insights into the etiology of insomnia, highlighting biological pathways that are shared with other complex disorders. Increased focus on treating sleep problems in the clinic and through public health interventions may reduce the overall burden of disease in human populations.

## Epidemiology of insomnia

Sleep plays a critical role in maintaining mental and physical health. Studies investigating the effects of short-term sleep restriction have noted elevated blood pressure, metabolic changes, increased inflammation and activation of the sympathetic nervous system [1]. Chronic poor sleep has been linked to a range of physical health problems such as heart disease, diabetes, and gastrointestinal problems [2]. The links between poor sleep and brain function are even more pronounced, with sleep disturbances being a common feature of many psychiatric and neurological disorders [3].

Insomnia is characterized by complaints about the quality or duration of sleep accompanied by significant daytime impairment. Occasional insomnia is reported by approximately 30% of the population, whereas insomnia disorder, which requires significant insomnia three nights per week for at least 3 months, has a prevalence of approximately 10% [4], making it a major public health concern. As it is comorbid with a range of health problems, insomnia is one of the commonly reported concerns in medical practice. Importantly, insomnia is also associated with decreased health-related quality of life and mortality [4].

## Genetic underpinnings of insomnia

Although the condition is common, the pathophysiological mechanisms of insomnia are not well understood. A substantial proportion of the risk of insomnia can be attributed to genetic variation [5]. Two recent large genome-wide association studies (GWAS) involving hundreds of thousands of individuals have yielded pivotal insights into the biological mechanisms underlying insomnia and its relationship with physical and mental health [6, 7], opening up a host of potential new avenues for research.

Although both studies leveraged the UK Biobank resource, they utilized slightly different definitions of insomnia. Lane and colleagues [6] conducted an analysis in which participants who reported occasional sleep difficulties were designated as cases, whereas Jansen and colleagues [7] denoted those with only occasional sleep difficulties as controls. Furthermore, Jansen and colleagues included data from the personalized genomics company 23andMe to yield a sample size of more than 1.3 million [7]. The prevalence of insomnia was 28% in the UK Biobank and 31% in the 23andMe cohort, similar to the prevalence of occasional insomnia in epidemiological studies.

A total of 248 independent single nucleotide polymorphisms (SNPs) in 202 genomic loci were identified by Jansen et al. [7], whereas Lane et al. [6] reported 57 independent associations in the UK Biobank. The overwhelming majority of these loci have not been previously implicated in the regulation of sleep in humans or model organisms. Importantly, the associations appear robust even when considering possible confounders such as body mass index (BMI), caffeine consumption and existing comorbidities.

### Genetic correlation of insomnia with complex traits

Genetic correlation analysis, which estimates the degree of correlation between the genetic influences on one trait and those on another, reveals significant genetic overlap between insomnia and a range of complex traits. Perhaps the most striking finding is that insomnia is negatively genetically correlated with longevity (genetic correlation, rG = − 0.32), as measured using parents’ age of death in UK Biobank. This provides compelling evidence of a genetic link between poor sleep and all-cause mortality, highlighting the importance of sleep in maintaining good health.

The pattern of observed genetic correlations refines our understanding of the nature of insomnia. Jansen and colleagues [7] show that the genetic correlations between insomnia and other self-reported sleep traits, with the exception of sleep duration (rG with insomnia = − 0.47), in the UK Biobank are modest. Given that insomnia complaints often relate to sleep of insufficient duration, it is perhaps not surprising that there is a strong negative genetic correlation with self-report sleep duration. A key question is what is the relationship between self-reported insomnia and objective measures of sleep such as sleep duration and frequency of awakenings. Objective measures of sleep are not used in the diagnosis of insomnia because many insomniacs show no evidence of abnormal sleep by polysomnography. Lane and colleagues [7] found that the identified genetic variants for insomnia are also associated with decreased sleep duration measured using accelerometer data in a subset of the UK Biobank sample, but much work remains to be done to evaluate the association of insomnia risk variants with objective sleep measures. The underlying mechanisms of insomnia and its comorbidities may be different in those who have objectively poor sleep in addition to self-report complaints [8].

#### Insomnia and psychiatric disorders

Genetic correlations were highest with psychiatric disorders such as depression (rG = 0.59), anxiety (rG = 0.56) and attention-deficit hyperactivity disorder (ADHD; rG = 0.45), indicating that the etiology of insomnia is similar to those of other psychiatric disorders. Insomnia forms part of the diagnostic criteria for depression and is the most commonly reported symptom of depression. Depression is the most commonly studied comorbidity of insomnia, with strong evidence of bidirectional effects between the two conditions. Anxiety and ADHD are disorders that are also often comorbid with sleep problems and depression, and they are characterized by hyperarousal, a key feature of insomnia. It was notable that the genetic correlations of insomnia with schizophrenia (rG = 0.13) and bipolar disorder (rG = 0.08) were much lower than those for other psychiatric disorders. Disruptions to sleep are common in both schizophrenia and bipolar disorder, and both are strongly correlated with depression, yet their genetic overlap with insomnia is low. Moreover, both disorders are significantly positively correlated with sleep duration [9], indicating that the genetic underpinnings of sleep disruption differ between psychiatric disorders and providing valuable clues into the etiological differences between these disorders that should be investigated further.

#### Insomnia and metabolic traits

Although weaker than the correlations with psychiatric disorders, there is also a correlation between genetic risk factors for insomnia and increased BMI (rG = 0.16), risk of type 2 diabetes (rG = 0.20) and risk of coronary artery disease (rG = 0.18). These results reinforce findings from studies showing that both acute and chronic sleep deprivation have profound metabolic consequences [1].

### Mendelian randomization

A significant limitation of the existing cross-sectional epidemiological studies is that they do not allow the inference about the direction of causation of sleep difficulties and comorbid health problems. Mendelian randomization (MR) is a statistical technique that uses genetic risk variants as instrumental variables to try to infer the direction of causality between two associated traits. Both of the GWAS discussed above found significant evidence that insomnia is a causal risk factor for metabolic traits (type 2 diabetes, BMI and coronary artery disease) and neuropsychiatric traits (depressive symptoms, subjective well-being and anxiety), with little evidence for effects in the other direction. Although MR does not definitively answer the question of causality and the relationships are likely to be complex, these results tilt the balance of evidence towards sleep as the driver of the association with mental and physical health problems. The implication of these findings is that interventions targeting insomnia may be effective in decreasing the burden of health problems in the population, an idea that is supported by the findings of a recent clinical trial [10]. At present, it is not clear how the estimated causal effects of insomnia on disease phenotypes can be compared directly because of differences in ascertainment and of the fractions of cases and controls in different genetic studies. The adoption of more commonly used measures of epidemiological risk will help to make the results more interpretable and will guide further clinical studies. It will also be important to further dissect these causal associations in order to gain deeper insight into the mechanisms.

## Concluding remarks

The large genetic studies discussed here are a substantial leap forward for the field of insomnia research. The implications for clinical practice are that problems with sleep may reflect an underlying psychiatric disorder or other complex disorder, and therefore the screening of patients with sleep problems is essential. Identifying the specific type of sleep problem may aid in the diagnosis of psychiatric disorders, given the contrasting patterns of genetic correlation of different psychiatric disorders with sleep difficulties. As technology improves, and as it becomes cost-effective to measure both subjective and objective sleep in a noninvasive way, information about sleep quality may prove invaluable in the diagnosis and monitoring of disease progression. Furthermore, interventions to improve sleep provide a novel target for disease treatment that should be investigated more widely.

There remains much more to be revealed about the biological pathways that lead to sleep problems. The identified variants explain only a small proportion of the overall risk, highlighting the need for even larger studies. The next steps should involve studies in diverse cohorts recruited in different settings, including patients with insomnia disorder, to further elucidate the role of sleep in maintaining good health. Addressing sleep difficulties represents a promising new frontier in the effort to reduce the burden of disease in the population.

## Abbreviations

ADHD: Attention-deficit hyperactivity disorder
BMI: Body mass index
GWAS: Genome-wide association study
MR: Mendelian randomization
